# Impact of Watercress Consumption on Antioxidant Defense and Oxidative Stress Among Adults with Different Levels of Exposure to Cigarette Smoke in Chiang Mai, Thailand

**DOI:** 10.3390/antiox14121466

**Published:** 2025-12-07

**Authors:** Puriwat Fakfum, Praporn Kijkuokool, Wason Parklak, Hataichanok Chuljerm, Chikondi Maluwa, Irina Stepanov, Kanokwan Kulprachakarn

**Affiliations:** 1 School of Health Sciences Research, Research Institute for Health Sciences, Chiang Mai University, Chiang Mai 50200, Thailand; puriwat_f@cmu.ac.th (P.F.); praporn_k@cmu.ac.th (P.K.); hataichanok.ch@cmu.ac.th (H.C.); chikondi_maluwa@cmu.ac.th (C.M.); 2 Research Center for Non-Infectious Diseases and Environmental Health, Research Institute for Health Sciences, Chiang Mai University, Chiang Mai 50200, Thailand; wason.p@cmu.ac.th; 3 Division of Environmental Health Sciences, School of Public Health, University of Minnesota, Minneapolis, MN 55455, USA; stepa011@umn.edu; 4 Institute for Global Cancer Prevention Research, Masonic Cancer Center, University of Minnesota, Minneapolis, MN 55455, USA

**Keywords:** watercress, cigarette smoke, antioxidant capacity, oxidative stress marker

## Abstract

Antioxidant intake from natural sources may enhance defense systems against oxidative stress induced by environmental factors such as cigarette smoke. Watercress (*Nasturtium officinale*) is an abundant source of antioxidants. This study aimed to determine the antioxidant effects of watercress consumption in people exposed to cigarette smoke in Chiang Mai, Thailand. Forty-five participants (15 non-smokers, 15 non-smokers with self-reported exposure to secondhand smoke (SHS), and 15 smokers) aged 20–60 years consumed 60 g of fresh watercress with three meals daily for seven days. Clinical characteristics, lipid profiles, total antioxidant capacity, and oxidative stress markers were evaluated before and after the intervention. After 7 days of watercress consumption, body mass index (BMI) and hip circumference significantly decreased among non-smokers. Watercress consumption reduced total cholesterol and low-density lipoprotein cholesterol (LDL-C) levels in smokers and in SHS-exposed non-smokers; however, high-density lipoprotein cholesterol (HDL-C) significantly decreased in SHS-exposed non-smokers. Catalase activity increased, and malondialdehyde levels decreased in all groups. One of the measures of plasma total antioxidant capacity significantly improved in non-smokers. These findings suggest that watercress consumption may improve lipid profiles and mitigate oxidative stress, and that these beneficial effects differ across people with different levels of exposure to cigarette smoke. However, further studies are suggested to clarify these results.

## 1. Introduction

Exposure to cigarette smoke, both direct and indirect, poses significant health risks to individuals. Tobacco smoke is a complex mixture containing more than 9500 chemical compounds, including hydrocarbons, oxygenated and nitrogenous compounds, and other miscellaneous constituents [1]. According to the U.S. Food and Drug Administration (FDA) list established in 2012, 93 of these compounds are classified as harmful or potentially harmful, with 79 identified as carcinogenic [2].

Direct exposure occurs through active smoking, where individuals inhale harmful chemicals and carcinogens directly from cigarettes or other tobacco products. Indirect exposure, also known as secondhand smoke, affects non-smokers who inhale smoke from burning tobacco products or exhaled smoke from smokers. Both forms of exposure can lead to various health issues, including respiratory problems, cardiovascular diseases, and an increased risk of cancer [3].

The complex mixture of chemicals in cigarette smoke contains numerous carcinogens and irritants that can trigger or exacerbate respiratory conditions such as bronchitis, pulmonary fibrosis, and chronic obstructive pulmonary disease (COPD) [4]. Moreover, exposure to cigarette smoke adversely affects the cardiovascular system by promoting endothelial dysfunction, increasing oxidative stress, and accelerating the development of atherosclerosis, thereby elevating the risk of heart attacks and strokes [5]. Vulnerable populations, such as children and pregnant women, are especially at risk of the harmful effects of secondhand smoke [6]. The health risks associated with cigarette smoke exposure are closely linked to their ability to promote oxidative stress through the excessive generation of reactive oxygen species (ROS) [7,8].

Under normal physiological conditions, cellular metabolism generates free radicals and ROS, including superoxide anion radical (O_2_^•−^), hydrogen peroxide (H_2_O_2_), hydroxyl radical (HO^•^), nitric oxide radical (NO^•^), and more, through redox reactions [8]. The overproduction of these free radicals can damage the structure of lipids, proteins, DNA, and RNA, thereby disrupting cellular functions [9]. Cigarette smoke can exacerbate this imbalance, overwhelming the body’s antioxidant defenses and inducing oxidative stress [7,8,9,10].

The human body typically possesses an antioxidant system to counteract the production of free radicals. However, a prolonged imbalance between free radicals and antioxidant levels results in oxidative stress, which is related to various health effects [11,12]. Dietary intake of antioxidants from natural sources, such as vitamin A, vitamin E, vitamin C, polyphenol compounds, carotenoids, zinc, copper, and selenium, can enhance our antioxidant systems [13].

Watercress (*Nasturtium officinale*) is a leafy cruciferous vegetable belonging to the Brassicaceae family. This plant is native to Asia, Europe, India, and Africa. This aquatic plant is typically cultivated in slow-moving water areas such as ponds, lakes, and canals [14]. In Thailand, watercress is abundantly planted in the southern regions [15]. Watercress is rich in glucosinolates and isothiocyanates (ITCs) and serves as a potential source of antioxidants, including phenolic acids, flavonoids, proanthocyanins, chlorophylls, lycopene, carotenoids, vitamins, and minerals [16]. In addition, a previous study found that watercress extract is rich in querce-tin-3-O-rutinoside and caffeic acid, which are phytochemicals involved in directly increasing antioxidant activity and therefore reducing free radicals [17]. Similarly, Clemente et al. [18] showed that watercress had a positive effect on protection against oxidative stress. According to a previous review, the consumption of watercress as a supplement may ameliorate chronic respiratory diseases, cardiovascular diseases, diabetes, and cancer [19]. Nevertheless, evidence of the effects of watercress consumption on antioxidant defense and detoxification capacity in individuals exposed to cigarette smoke remains unclear.

This study aimed to investigate the antioxidant capacity of watercress consumption in adults exposed to different levels of cigarette smoke in Chiang Mai, Thailand. Findings from this study will contribute to a more comprehensive understanding of the potential protective role of watercress against oxidative stress induced by environmental and lifestyle-related exposures.

## 2. Materials and Methods

### 2.1. Study Population and Ethical Approval

Healthy subjects, both male and female (not pregnant or breastfeeding), aged between 20 and 60 years from Chiang Mai province were selected to participate in the study. Three groups of participants were recruited (n = 15 each): a non-smoker group (<100 cigarettes over a lifetime and none within the past 1 year prior to screening), a non-smoker exposed to secondhand smoke (SHS) group (exposed to SHS at home or at work at least once a week), and a smoker group (≥5 cigarettes/day for at least 1 year and not planning to quit). This study was approved by the Human Experimentation Committee at the Research Institute for Health Sciences, Chiang Mai University (Project No. 8/67) and Human Research Protection Program, University of Minnesota, USA (IRB ID: STUDY00022875). Written informed consent was obtained from all participating subjects.

### 2.2. Study Design and Blood Sample Collection

At baseline, demographic data, surveys on SHS exposure and smoking behavior, and medical history were collected from each subject. Blood samples were collected in heparinized tubes after an overnight fast for laboratory measurements. Physical examination, vital signs, and exhaled carbon monoxide (CO) [CO > 8 parts per million (ppm) as an indicator of active smoking] were also measured.

**Figure 1:**
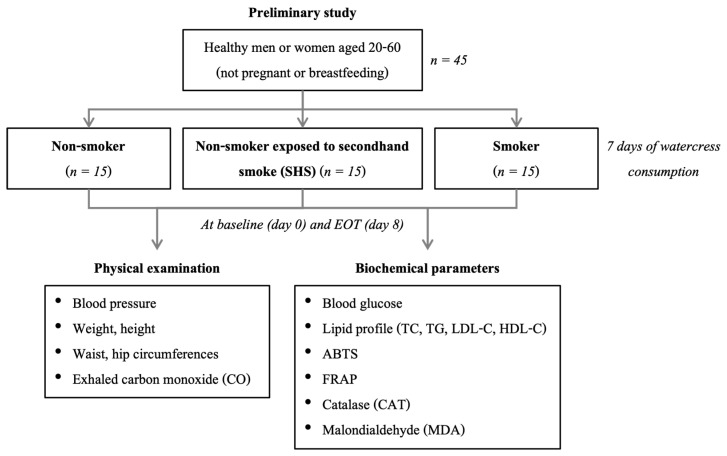
Study design of the current study. Abbreviations: EOT, end of treatment (watercress consumption); TC, total cholesterol; TG, triglyceride; LDL-C, low-density lipoprotein cholesterol; HDL-C, high-density lipoprotein cholesterol; ABTS, 2,2′-azino-bis-(3-ethylbenzothiazoline-6-sulfonic) acid; FRAP, ferric reducing antioxidant power.

During the intervention period, participants consumed 60 g of fresh watercress with each of three daily meals for seven consecutive days, following the amount used in the prior study [20]. Participants were also instructed to avoid other cruciferous vegetables throughout the intervention. At the end of the treatment phase, blood samples were again collected for laboratory measurements, biochemical analyses, and comprehensive physical assessments including measurements of vital signs and exhaled carbon monoxide were performed again to evaluate post-intervention changes. The study design is presented in Figure 1.

### 2.3. Blood Sample Preparation

Clotted blood tubes were used to assess fasting blood glucose (FBG) and serum-based biomarkers. FBG levels were determined from whole blood using an automated biochemistry analyzer (Rx Daytona, Randox Laboratories Ltd., Antrim, UK). Serum was separated by allowing the samples to clot and subsequently centrifuging them for lipid profile analysis using the same automated biochemistry analyzer. Plasma was obtained from blood samples collected in heparinized tubes by centrifugation at 3000× *g* for 10 min. The resulting plasma was used for the assessment of biochemical parameters, oxidative stress biomarkers, and total antioxidant capacity.

### 2.4. Antioxidant Capacity and Biomarker of Oxidative Stress Analysis

The antioxidant capacity assessed by 2,2′-azino-bis-(3-ethylbenzothiazoline-6-sulfonic) acid (ABTS) and ferric reducing antioxidant power (FRAP) assays and biomarkers of oxidative stress, including catalase (CAT) activity and malondialdehyde (MDA), were measured and analyzed in the plasma of all subjects at baseline (day 0) and after 7 days of watercress consumption.

#### 2.4.1. Plasma Total Antioxidant Capacity (FRAP Method and ABTS Enzyme Method)

Plasma total antioxidant capacity was evaluated using the colorimetric kit, including the ABTS enzyme method (Elabscience, Houston, TX, USA) and the FRAP method (Servicebio, Wuhan, Hubei, China). The principle of the ABTS method for determining the total antioxidant capacity (TAC) is as follows. The TAC of the plasma was evaluated and calculated by measuring the absorbance of the oxidized form of ABTS at 734 nm using a spectrophotometer (BMG LABTECH, Ortenberg, Germany). The principle of the FRAP method is that under acidic conditions, antioxidants can reduce Fe^3+^-triphenyltriazine (Fe^3+^-TPTZ) to produce blue Fe^2+^-TPTZ. The total antioxidant capacity of the sample was calculated by reading the absorbance at 590 nm using a spectrophotometer. The values were expressed in millimoles per liter (mmol/L).

#### 2.4.2. Determination of Oxidative Stress Markers by CAT Activity and MDA in Plasma Samples 

The CAT activity in the plasma samples from all subjects was measured using the commercial kit (Elabscience, Houston, TX, USA) according to the operating manual. The reaction that CAT breakdown H_2_O_2_ for 5 min was stopped by adding ammonium molybdate, the absorbance of the complex formed by the reaction of the residual H_2_O_2_ with ammonium molybdate was measured at 405 nm. The determinations were expressed in U/mL.

MDA levels in the plasma were measured by the MDA colorimetric assay kit (TBA method) (Elabscience, Houston, TX, USA). MDA was allowed to react with thiobarbituric acid (TBA), and the absorption of the formed complex was measured at 532 nm. The determinations were expressed in micromoles per liter (μmol/L).

### 2.5. Statistical Analysis

Descriptive statistics were used to summarize the demographic and baseline characteristics of the participants. Non-normally distributed paired variables including biomarkers, antioxidant capacity, smoking status, exhaled CO levels, FBG, lipid profiles, and physical examination findings were analyzed using the Wilcoxon matched-pairs signed-rank test. The Kruskal–Wallis test was employed to compare all variables across the three exposure groups. Categorical variables, such as SHS exposure status and frequency of exposure, were compared using Pearson’s chi-squared test or Fisher’s exact test, as appropriate. All statistical analyses were performed using Stata software (version 17; StataCorp LLC, College Station, TX, USA), and were considered significant at a *p*-value < 0.05.

## 3. Results

### 3.1. Baseline Demographic Characteristics

The baseline characteristics of study participants are presented in Table 1. A total of 45 participants were included in the study, divided equally into three groups: the non-smoker group (n = 15), the SHS-exposed non-smoker group (n = 15), and the smoker group (n = 15). The average age of participants was 36.7 ± 10.5 years, with similar age distributions across groups. Among all participants, 26 participants (57.8%) were male, and 19 participants (42.2%) were female, with a higher proportion of males in the smoker group (86.7%) compared to other groups. Most participants had an education level above high school (77.8%). Over half of the participants (51.1%) were employees. The associations between watercress consumption and selected covariates, including exercise behavior and medication use, are presented in Tables S1 and S2. No statistically significant differences were observed for these variables.

### 3.2. Smoking Behavior, SHS Exposure, and Exhaled CO Levels

Table 2 presents the characteristics of smoking behavior and SHS exposure. Among the current smokers, the median (interquartile range (IQR)) smoking duration at baseline was 10.54 (6.77–12.28) years. The number of cigarettes smoked per day was 10 (5–10) at baseline and 7 (5–12) after 7 days of watercress consumption. There were no significant differences in the proportion of SHS exposure and the frequency of exposure per week between baseline and after 7 days of watercress consumption in the SHS-exposed non-smoker group. The level of exhaled CO did not significantly change between visits. Additionally, smokers had a median duration of smoking of 10.51 years (IQR = 6.77–12.28).

In the SHS-exposed non-smoker group, exhaled CO levels showed no significant change after 7 days of watercress consumption, with the median (IQR) of 3 (2–3) ppm at baseline and 2 (2–3) ppm at the end of the consumption period. Similarly, there was no significant difference among non-smokers, with median (IQR) values of 2 (2–4) ppm at baseline and 2 (2–3) ppm at the end of the consumption period. Among smokers, exhaled CO levels were elevated but did not differ significantly between baseline (median = 16 ppm, IQR = 9–25) and the end of the watercress consumption period (median = 17 ppm, IQR = 9–27).

### 3.3. Physical Examination and Blood Pressure Results

Table 3 presents a comparison of physical examinations of participants grouped into non-smoker, SHS-exposed non-smoker, and smoker groups before and after watercress consumption. No significant differences were observed in changes in height, body weight, and waist circumference before and after watercress consumption across all groups. Interestingly, among the non-smoker group, body mass index (BMI) showed a significant change after 7 days of watercress consumption, with a median (IQR) of 24.8 (21.2–27.7) at baseline and 24.5 (21.2–27.7) at the end of watercress consumption (*p* = 0.012). Hip circumference of the non-smoker group was also significantly higher after watercress consumption, median (IQR) = 96 (88–103), compared to the baseline value, median (IQR) = 94.5 (91–104), with a *p*-value of 0.042. Heart rate and blood pressure (both systolic and diastolic) values showed no statistically significant differences; however, diastolic blood pressure tended to decrease after watercress consumption. Moreover, comparisons of physical examination results and blood pressure values among the three groups showed no significant differences at baseline or at the end of the watercress consumption period.

### 3.4. Comparison of Biochemical Parameters

Table 4 presents the biochemical data for the groups of non-smokers, SHS-exposed non-smokers, and smokers, both prior to and following the period of watercress consumption. Among the blood biochemical parameters, FBG did not exhibit significant changes after watercress consumption in any group. However, the median of FBG tended to decrease from 91 mg/dL to 89 mg/dL in the non-smoker group; from 94 mg/dL to 89 mg/dL in the SHS-exposed non-smoker group; and from 91 mg/dL to 87 mg/dL in the smoker group.

The lipid profiles, including total cholesterol (TC), triglyceride (TG), low-density lipoprotein cholesterol (LDL-C), and high-density lipoprotein cholesterol (HDL-C), showed no significant changes in the non-smoker group before and after the watercress consumption period. In contrast, the SHS-exposed non-smoker group demonstrated significant changes in all lipid parameters except TG. Specifically, TC in the SHS-exposed non-smoker group significantly decreased after watercress consumption (median = 190 mg/dL, IQR = 159–216) compared to the baseline level (median = 230 mg/dL, IQR = 199–244), with a *p*-value of less than 0.001. Similarly, LDL-C significantly decreased post-watercress consumption (median = 119 mg/dL, IQR = 93–182) compared to the baseline level (median = 167 mg/dL, IQR = 109–197), with a *p*-value of 0.017. HDL-C also significantly decreased after watercress consumption (median = 39.61 mg/dL, IQR = 30.96–51.97) compared to the baseline level (median = 45.46 mg/dL, IQR = 42.11–56.13), with a *p*-value of 0.003. Among smoker group, TC significantly decreased after watercress consumption (median = 190 mg/dL, IQR = 146–213) compared to the baseline level (median = 225 mg/dL, IQR = 187–253), with a *p*-value of 0.025. Similarly, LDL-C significantly decreased post-watercress consumption (median = 143 mg/dL, IQR = 104–174) compared to the baseline level (median = 174 mg/dL, IQR = 143–224), with a *p*-value of 0.019. Although the medians of TG and HDL in the smoker group tended to decrease after watercress consumption, from 129 mg/dL to 114 mg/dL and from 39.14 mg/dL to 34.91 mg/dL, respectively, these changes were not statistically significant. In addition, comparisons of biochemical parameter results among the three groups showed no significant differences at baseline or at the end of the watercress consumption period.

### 3.5. Analysis of Antioxidant Capacity and Oxidative Stress Markers

**Figure 2:**
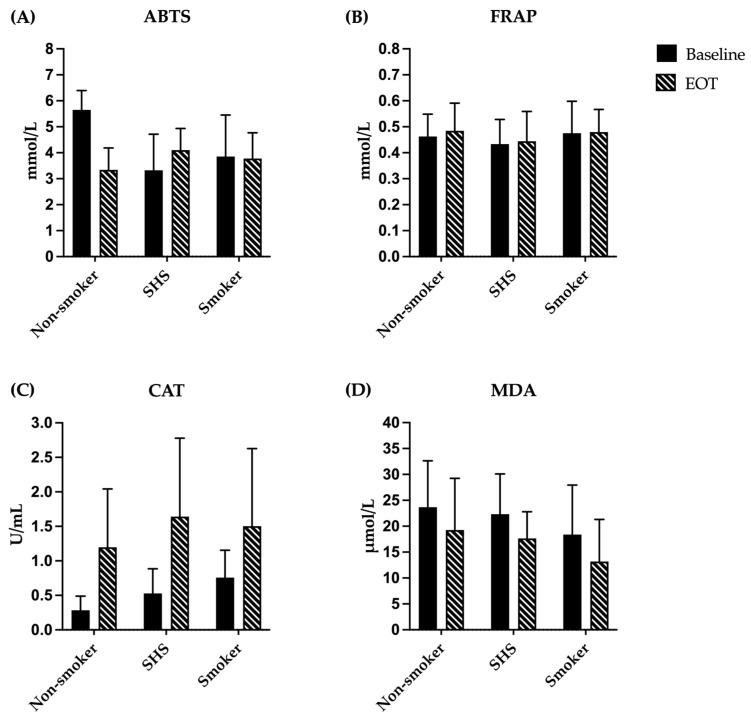
Antioxidant and oxidative stress markers of participants in non-smoker, SHS-exposed non-smoker, and smoker groups. (**A**) ABTS level and (**B**) FRAP were expressed as mmol/L; (**C**) CAT level was expressed as U/mL; and (**D**) MDA level was expressed as μmol/L. Data are mean ± SD (n = 15 each). Abbreviations: ABTS, 2,2′-azino-bis-(3-ethylbenzothiazoline-6-sulfonic) acid; FRAP, ferric reducing antioxidant power; CAT, catalase; MDA, malondialdehyde; EOT, end of treatment (watercress consumption); SHS, non-smoker exposed to secondhand smoke.

The antioxidant capacity and oxidative stress markers, including ABTS, FRAP, CAT, and MDA, among participants in the non-smoker, SHS-exposed non-smoker, and smoker groups are presented in Table 5 and Figure 2. Following the consumption of watercress, the median ABTS level significantly decreased in the non-smoker group from 5.91 mmol/L (IQR = 5.17–6.10) to 3.54 mmol/L (IQR = 2.65–4.15), with a *p*-value of less than 0.001. However, no significant difference in ABTS was observed between pre- and post-watercress consumption in the SHS-exposed non-smoker and smoker groups. The FRAP antioxidant capacity test exhibited no significant change after watercress consumption in any group.

Regarding CAT activity, watercress consumption significantly enhanced the CAT level in all groups: from a median of 0.24 U/mL (IQR = 0.15–0.39) to a median of 0.90 U/mL (IQR = 0.48–2.00) in the non-smoker group, with a *p*-value of 0.016; from a median of 0.39 U/mL (IQR = 0.34–0.71) to a median of 1.82 U/mL (IQR = 0.42–2.57) in the SHS-exposed non-smoker group, with a *p*-value of 0.020; and from a median of 0.78 U/mL (IQR = 0.56–0.83) to a median of 1.52 U/mL (IQR = 0.34–2.67) in the smoker group, with a *p*-value of 0.039. Conversely, the MDA level, an indicator of lipid peroxidation, significantly decreased after watercress consumption across all groups. The median MDA level in the non-smoker group declined from 24.67 μmol/L (IQR = 18.37–29.11) to 15.41 μmol/L (IQR = 9.85–29.85), with a *p*-value of 0.041. Likewise, in the SHS-exposed non-smoker group, the median MDA level decreased from 23.19 μmol/L (IQR = 14.67–28.37) to 17.26 μmol/L (IQR = 15.04–19.48), with a *p*-value of 0.040. The smoker group also demonstrated a significant reduction, with median MDA levels decreasing from 15.41 μmol/L (IQR = 10.59–26.89) to 12.44 μmol/L (IQR = 6.52–19.48), with a *p*-value of 0.006. At baseline, significant differences among the three groups were observed in the ABTS assay and CAT activity (*p* < 0.01), with the SHS-exposed non-smoker group showing lower antioxidant capacity by the ABTS assay and the non-smoker group exhibiting lower CAT levels. However, after 7 days of watercress consumption, no significant differences were found among the groups in all antioxidant and oxidative stress markers.

## 4. Discussion

Cigarette smoke is one of the major environmental risk factors contributing to the development of various non-communicable diseases (NCDs) [3]. Exposure to cigarette smoke can induce oxidative stress and lead to adverse health outcomes [8,9,10,11,12]. Watercress contains bioactive compounds that act as antioxidants [16]. In addition, watercress consumption has the potential to ameliorate NCDs such as cardiovascular diseases and diabetes [19]. The current study investigated the antioxidant capacity and oxidative stress marker of watercress consumption on Thai adults exposed to cigarette smoke, particularly in Chiang Mai province, through analysis of before- and after-intervention outcomes of biochemical and antioxidant parameters.

At baseline, the physical examination parameters, blood pressure, and heart rate were comparable across all groups, indicating that participants were generally similar despite being exposed to different levels of cigarette smoke. After the 7-day watercress consumption period, no significant differences were observed between groups.

When analyzed within each group, significant changes in physical examination parameters were observed among non-smokers, showing a trend toward decreased BMI. Previous studies have suggested that watercress may contribute to weight control and body composition reduction by suppressing dietary sugar and fat absorption [21]. However, there is still no clear evidence in humans confirming that fresh watercress consumption directly reduces body composition or body weight. Our findings indicate that a 7-day consumption of watercress may help improve body composition, particularly among healthy individuals without smoke exposure.

Regarding blood biochemical parameters, baseline FBG and lipid profiles across the three participant groups, non-smokers, SHS-exposed non-smokers, and smokers, were not significantly different. Similarly, at the end of the intervention, no significant differences were observed among the groups. However, watercress consumption produced different responses within each group. Notably, FBG did not exhibit significant changes in any group following watercress consumption. However, a trend towards reduced FBG levels was observed across all groups.

Although previous studies have demonstrated that watercress consumption significantly lowers blood glucose levels in diabetes-induced rats, evidence from human studies remains limited. For example, Hadjzadeh et al. [22] administered watercress extract (100 and 200 mg/kg/day) to diabetic female Wistar rats over a four-week period, resulting in a significant reduction in serum glucose levels compared to the diabetic control group. Nonetheless, the anti-diabetic effects of watercress consumption in humans remain unclear. For instance, Shokraei et al. [23] conducted a randomized crossover trial among healthy young men (n = 16). They investigated the effects of adding either romaine lettuce or watercress (100 g each) to a moderately high-fat meal on postprandial glucose and insulin responses. The results indicated that consumption of lettuce, but not watercress, significantly reduced the postprandial glucose area under the curve (AUC) compared to the control meal. Similarly, no significant difference in insulin AUC was observed between the watercress-added and control meal groups.

Watercress consumption appeared to exert stronger effects on lipid profiles among SHS-exposed non-smokers and smokers, particularly TC and LDL-C, suggesting potential cardiovascular benefits. Bahramikia and Yazdanparast [24] demonstrated that watercress improves lipid profiles in an animal model. Hypercholesterolemic rats were treated with a hydroalcoholic extract of watercress at a dose of 500 mg/kg/day. After ten days of treatment, there was a significant reduction in TC, TG, and LDL-C, while HDL-C was significantly higher compared to untreated hypercholesterolemic rats. A clinical study conducted by Clemente et al. [25] also revealed that the consumption of watercress extract (dose of 750 mg/kg/day) significantly improved LDL-C levels among overweight individuals with physical disabilities. Consistent with these findings, the decreases in TC and LDL-C observed in our study support the hypothesis that fresh watercress can modulate lipid metabolism. Possible mechanisms include increased bile acid binding and excretion, resulting in reduced intestinal cholesterol absorption, as well as inhibition of endogenous cholesterol biosynthesis and upregulation of LDL receptors [14].

Interestingly, HDL-C levels tended to decrease in SHS-exposed non-smokers and smokers after watercress intake. Smoking is known to alter lipid metabolism [26,27]. The possible mechanism is that oxidative damage caused by cigarette smoke destroys HDL particles, impairing their antioxidant properties and reducing lipid transport and atheroprotective capabilities [28].

In contrast, non-smokers exhibited no significant changes in lipid profiles after watercress consumption. This highlights the complex nature of lipid regulation and suggests that the effects of dietary interventions may differ according to smoking exposure. The bioactive phytochemicals in watercress, such as phenolic and flavonoid, may contribute to the lipid-lowering effects through preventing formation of free radicals, reducing oxidative stress, and inhibiting lipid peroxidation [29,30], particularly under conditions of increased oxidative stress, such as cigarette smoke exposure. Non-smokers without such oxidative stress may therefore exhibit less pronounced metabolic responses to watercress consumption. Moreover, a previous clinical study reported that watercress consumption exhibits strong anti-inflammatory effects by downregulating pro-inflammatory cytokines after exercise compared to the pre-exercise levels. The levels of inflammatory markers, such as interleukin-1 beta (IL-1β), interleukin-6 (IL-6), interleukin-1 (IL-10), and tumor necrosis factor-alpha (TNF-α), were even lower than the pre-exercise levels [31]. This aligns with our findings, further supporting the hypothesis that watercress may exert protective effects in individuals exposed to oxidative stress from cigarette smoke.

For the antioxidant capacity, watercress is a notable source of phytochemical compounds, including flavonoids, vitamin C, and phenolic acids, which contribute to enhancing antioxidant capacity and reducing oxidative stress [19]. Previous studies have reported that watercress supplementation may reduce oxidative stress and increase antioxidant capacity. For example, Clemente et al. [25,32] demonstrated that consumption of watercress leaf extracts led to decreased MDA levels, a biomarker of oxidative stress. Although these studies used extracts, our findings using fresh watercress also led to significant reductions in MDA levels across all groups. This observation is consistent with our lipid profile results, which showed that after fresh watercress consumption, TC and LDL-C levels decreased, and these changes were associated with plasma MDA concentrations [33]. Together, these findings suggest that fresh watercress consumption may effectively reduce oxidative stress. In addition, Clemente et al. [32] also found that consuming watercress leaf extracts observed an increase in antioxidant capacity by increasing activity of the enzymatic antioxidant CAT. Research by Gill et al. [34] demonstrated that consuming 85 g of raw watercress increases plasma antioxidant concentrations of β-carotene and lutein. However, to date, no clinical study has assessed the effect of acute raw watercress consumption on plasma antioxidant capacity through the determination of CAT activity. Therefore, for the first time, our study found that CAT activity was significantly higher after 7 days of watercress consumption in all groups. Although, in the present study, at baseline, CAT activity in the non-smoker group was lower than that in smokers, which is contrary to previous finding reporting decreased catalase activity in smokers compared to non-smokers [35]. This unexpected difference may be related to individual variations in lifestyle or diet. However, after 7 days of watercress consumption, CAT activity significantly increased in all groups. This finding suggests that acute raw watercress consumption may enhance the activity of antioxidant enzymes, including CAT. The observed reduction in MDA and the increase in CAT activity is inversely related, reflecting improved antioxidant capacity and reduced lipid peroxidation. Watercress contains bioactive compounds that enhance antioxidant enzyme activity, including CAT, by increasing the activity of the nuclear factor erythroid 2-related fac-tor 2 (Nrf2) pathway, thereby lowering MDA levels [36].

Our study did not observe a significant increase in total antioxidant capacity markers, including FRAP and ABTS assays, after watercress consumption. Previous studies that reported changes in FRAP levels following watercress intake used concentrated extracts and had longer intervention durations than ours [19,37]. Therefore, consuming fresh watercress for only 7 days at a dose of 60 g per meal may not be sufficient to increase total antioxidant capacity by FRAP assay. Moreover, we observed a statistically significant decrease in plasma antioxidant capacity measured by the ABTS assay following consumption of watercress in the non-smoker group. In addition, comparison of the ABTS assay results among the groups showed that the SHS-exposed non-smoker group had significantly lower values than other groups. These results are unexpected given prior reports that watercress consumption increases plasma antioxidant markers in humans and in vitro. However, several methodological and biological explanations can reconcile our findings. For example, the report by Schaich et al. [38] showed the ABTS assay has limitations for the quantitative evaluation of antioxidant capacity in natural materials or biological tissues. They suggested that it should be replaced by other methods with clearly identifiable reaction mechanisms, fully tested reaction conditions, and kinetic analyses, including lipid oxidation assays. In addition, pre-analytical and physiological confounders such as pre-sampling preparation of participants, prior diet, physical activity stress, and sleep may influence the ABTS assay. These factors may partly explain the unexpected decrease in ABTS assay observed after watercress consumption.

Despite the promising findings, some potential confounding factors were observed during treatment such as the dietary intake, exercise behavior and medication use. Throughout the study, participants were instructed to maintain their usual eating habits and to avoid consumption of other cruciferous vegetables. A brief dietary diary was recorded three days before starting the watercress consumption period, during the intervention, and one week after the intervention. No obvious changes in overall dietary pattern were identified. Participants mainly consumed watercress as salad, smoothies, sandwich, or side dish. However, the quantity of food intake was not recorded; therefore, the total energy intake, macronutrients, and micronutrients could not be estimated. Additionally, the analysis of the association between watercress consumption and the covariates, including exercise behavior and medication use. No statistically significant associations were observed, as presented in the Supplementary Materials.

Several limitations of this study should also be acknowledged. As a preliminary investigation and the first of its kind conducted in Thailand, the sample size was relatively small and based on a similar previous study rather than a formal sample size calculation. In addition, the proportion of male and female participants was unbalanced. The duration of watercress consumption was limited to seven days, which might not have been long enough to fully observe long-term biochemical and physiological changes. In addition, there is no control group or placebo condition in the study. Regarding the intervention material, no chemical characterization of the watercress was performed; therefore, the specific bioactive compounds and their quantities were not identified. Moreover, participants were classified by their smoking exposure using exhaled CO levels and self-reported smoke exposure history. However, no additional biomarkers of tobacco exposure, such as nicotine or cotinine levels, were measured.

Future research should therefore include urinary cotinine measurements for more accurate classification of smoke exposure. Larger, more balanced, and statistically powered sample sizes are needed to confirm these findings. Additionally, chemical profiling of the watercress and the quantification of key phytochemicals would give a clearer understanding of the dose–response relationship. Furthermore, it would also be beneficial to investigate the molecular and cellular mechanisms underlying the observed effects, particularly the modulation of oxidative stress pathways and inflammatory signaling in populations chronically exposed to cigarette smoke.

## 5. Conclusions

In conclusion, this research provides new evidence that short-term consumption of fresh watercress may enhance antioxidant defenses and lessen oxidative stress in adults exposed to cigarette smoke. After a week of watercress consumption, CAT activity significantly increased, while MDA levels markedly decreased across all participant groups, indicating an improved oxidative balance. Additionally, reductions in TC and LDL-C levels observed among smokers and SHS-exposed non-smokers suggest that watercress may offer cardiovascular protective benefits, especially for individuals exposed to cigarette smoke. These results imply that adding fresh watercress to the diet could help reduce oxidative stress and enhance metabolic health in populations exposed to tobacco smoke. However, further studies with larger sample sizes and extended intervention durations are necessary to confirm these effects and to clarify the underlying biochemical pathways.

## Figures and Tables

**Table 1:** Demographic characteristics of participants (n = 45).

Characteristic	Non-Smoker (n = 15)	SHS (n = 15)	Smoker (n = 15)	Total (n = 45)
Age (years), Mean ± SD	36.47 ± 9.96	35.2 ± 9.74	38.4 ± 12.12	36.69 ± 10.5
Gender, n (%)				
Male	8 (53.3)	5 (33.3)	13 (86.7)	26 (57.8)
Female	7 (46.7)	10 (66.7)	2 (13.3)	19 (42.2)
Education, n (%)				
Intermediate school	0 (0.0)	0 (0.0)	2 (13.3)	2 (4.4)
Junior high school	1 (6.7)	2 (13.3)	5 (33.3)	8 (17.8)
High school/vocational	2 (13.3)	3 (20.0)	5 (33.3)	10 (22.2)
Bachelor’s degree	4 (26.7)	9 (60.0)	3 (20.0)	16 (35.6)
Master’s degree	5 (33.3)	1 (6.7)	0 (0.0)	6 (13.3)
PhD degree	3 (20.0)	0 (0.0)	0 (0.0)	3 (6.7)
Occupation, n (%)				
Student	2 (13.3)	1 (6.7)	0 (0.0)	3 (6.7)
Employee	6 (40.0)	8 (53.3)	9 (60.0)	23 (51.1)
Government agencies	6 (40.0)	4 (26.7)	2 (13.3)	12 (26.7)
Self-employed	1 (6.7)	2 (13.3)	3 (20.0)	6 (13.3)
Other/housewife	0 (0.0)	0 (0.0)	1 (6.7)	1 (2.2)

Abbreviations: SHS, non-smoker exposed to secondhand smoke.

**Table 2:** Smoking behavior, SHS exposure, and level of exhaled CO in participants consuming watercress.

	Non-Smoker (n = 15)			SHS (n = 15)			Smoker (n = 15)		
	Baseline	EOT	*p*-Value	Baseline	EOT	*p*-Value	Baseline	EOT	*p*-Value
Currently smoking, %	0%	0%	-	0%	0%	-	100%	100%	-
Duration of smoking (years), Median (IQR)	-	-	-	-	-	-	10.51 (6.77–12.28)	-	-
Cigarettes per day, Median (IQR) ^a^	-	-	-	-	-	-	10 (5–10)	7 (5–12)	0.203
Exposed to SHS (yes), % ^b^	0%	0%	-	100%	93%	1.000	0%	0%	-
Frequency of SHS exposure (per week) ^b^	-	-	-	-	-	0.918	-	-	-
1 time, %	-	-	-	6.7%	7%		-	-	-
2–5 times, %	-	-	-	46.7%	36%		-	-	-
6–10 times, %	-	-	-	40%	50%		-	-	-
>10 times, %	-	-	-	6.7%	7%		-	-	-
Level of exhaled CO (ppm), Median (IQR) ^a^	2 (2–4)	2 (2–3)	0.699	3 (2–3)	2 (2–3)	0.109	16 (9–25)	17 (9–27)	0.423

Abbreviations: EOT, end of treatment (watercress consumption); SHS, non-smoker exposed to secondhand smoke; IQR, interquartile range; CO, carbon monoxide; ppm, part per million. ^a^ Analyzed by Wilcoxon signed-rank test. ^b^ Analyzed by Pearson Chi-square/Fisher’s exact.

**Table 3:** Comparison of physical examination and blood pressure of participants in non-smoker, SHS-exposed non-smoker, and smoker groups.

Characteristic	Watercress Consumption	Non-Smoker (n = 15)	SHS (n = 15)	Smoker (n = 15)	*p*-Value ^b^
		Median (IQR)	Median (IQR)	Median (IQR)	
Height (cm)	Baseline	162.0 (156.0–170.0)	164.5 (150.0–170.0)	170.0 (160.0–176.0)	0.190
	EOT	163.0 (156.0–170.0)	164.5 (150.0–170.0)	170.0 (160.0–176.0)	0.216
	*p*-value ^a^	1.00	1.00	1.00	
Weight (kg)	Baseline	61.50 (54.25–77.40)	68.00 (53.75–73.50)	65.90 (61.00–74.90)	0.703
	EOT	61.60 (54.70–76.60)	68.00 (53.75–73.10)	65.70 (60.55–75.70)	0.748
	*p*-value ^a^	0.445	0.480	0.382	
BMI (kg/m^2^)	Baseline	24.8 (21.2–27.7)	25.4 (21.0–28.4)	23.5 (21.6–28.4)	0.995
	EOT	24.5 (21.2–27.7)	25.0 (21.2–28.6)	23.4 (21.7–28.3)	0.980
	*p*-value ^a^	0.012 *	0.553	0.558	
Waist Circumference (cm)	Baseline	80.0 (69.0–93.0)	81.0 (72.0–91.0)	83.0 (76.0–92.0)	0.646
	EOT	81.0 (74.0–91.6)	86.0 (70.0–90.0)	83.0 (81.0–89.6)	0.610
	*p*-value ^a^	0.836	1.000	0.570	
Hip Circumference (cm)	Baseline	94.5 (91.0–104.0)	98.0 (92.0–106.5)	96.0 (90.5–101.0)	0.682
	EOT	96.0 (88.0–103.0)	97.0 (92.0–104.0)	96.0 (91.0–99.8)	0.643
	*p*-value ^a^	0.042 *	0.213	0.690	
Systolic blood pressure (mmHg)	Baseline	117 (100–127)	125 (109–128)	121 (113–133)	0.480
	EOT	117 (113–122)	120 (114–125)	124 (110–128)	0.811
	*p*-value ^a^	0.649	0.698	0.516	
Diastolic blood pressure (mmHg)	Baseline	82 (68–89)	85 (70–91)	80 (74–90)	0.715
	EOT	82 (70–85)	81 (72–88)	82 (71–88)	0.997
	*p*-value ^a^	0.606	0.053	0.212	
Heart rate (bpm)	Baseline	77 (67–83)	72 (64–81)	74 (69–79)	0.577
	EOT	73 (63–81)	73 (68–81)	75 (67–88)	0.855
	*p*-value ^a^	0.217	0.934	0.224	

Abbreviations: EOT, end of treatment (watercress consumption); SHS, non-smoker exposed to secondhand smoke; IQR, interquartile range; BMI, body mass index. ^a^ The comparison between baseline data and EOT data in each group was analyzed by the Wilcoxon signed-rank test. ^b^ The comparison across three groups was analyzed by the Kruskal–Wallis test. * Significantly different (*p*-value < 0.05).

**Table 4:** Comparison of biochemical parameters of participants in non-smoker, SHS-exposed non-smoker, and smoker groups.

Biochemical Parameters	Watercress Consumption	Non-Smoker (n = 15)	SHS (n = 15)	Smoker (n = 15)	*p*-Value ^b^
		Median (IQR)	Median (IQR)	Median (IQR)	
Fasting blood glucose, FBG (mg/dL)	Baseline	91 (86–104)	94 (84–102)	91 (88–94)	0.600
	EOT	89 (84–104)	89 (87–98)	87 (78–92)	0.214
	*p*-value ^a^	0.656	0.324	0.075	
Total cholesterol, TC (mg/dL)	Baseline	209 (170–242)	230 (199–244)	225 (187–253)	0.567
	EOT	206 (176–229)	190 (159–216)	152.1–207.76	0.345
	*p*-value ^a^	0.525	<0.001 *	0.025 *	
Triglyceride, TG (mg/dL)	Baseline	92 (74–114)	104 (78–160)	129 (77–167)	0.223
	EOT	83 (61–137)	98 (54–135)	114 (71–156)	0.521
	*p*-value ^a^	0.772	0.258	0.283	
Low-density lipoprotein cholesterol, LDL-C (mg/dL)	Baseline	139 (136–174)	167 (109–197)	174 (143–224)	0.278
	EOT	146 (135–166)	119 (93–182)	143 (104–174)	0.603
	*p*-value ^a^	0.561	0.017 *	0.019 *	
High-density lipoprotein cholesterol, HDL-C (mg/dL)	Baseline	44.37 (33.94–50.48)	45.46 (42.11–56.13)	39.14 (34.40–49.83)	0.143
	EOT	44.04 (30.15–48.03)	39.61 (30.96–51.97)	34.91 (28.39–39.68)	0.066
	*p*-value ^a^	0.847	0.003 *	0.055	

Abbreviations: EOT, end of treatment (watercress consumption); SHS, non-smoker exposed to secondhand smoke; IQR, interquartile range. ^a^ The comparison between baseline data and EOT data in each group was analyzed by the Wilcoxon signed-rank test. ^b^ The comparison across three groups was analyzed by the Kruskal–Wallis test. * Significantly different (*p*-value < 0.05).

**Table 5:** Comparison of antioxidant and oxidative stress markers of participants in non-smoker, SHS-exposed non-smoker, and smoker groups.

Biomarkers	Watercress Consumption	Non-Smoker (n = 15)	SHS (n = 15)	Smoker (n = 15)	*p*-Value ^b^
		Median (IQR)	Median (IQR)	Median (IQR)	
ABTS radical cation decolorization assay, ABTS (mmol/L)	Baseline	5.91 (5.17–6.10)	3.54 (2.59–4.72)	4.60 (3.13–4.83)	0.001 *
	EOT	3.54 (2.65–4.15)	4.14 (3.32–4.84)	4.11 (3.21–4.37)	0.108
	*p*-value ^a^	<0.001 *	0.083	0.561	
Ferric reducing antioxidant power, FRAP (mmol/L)	Baseline	0.44 (0.40–0.54)	0.43 (0.36–0.53)	0.45 (0.41–0.52)	0.765
	EOT	0.49 (0.41–0.58)	0.39 (0.36–0.54)	0.49 (0.38–0.53)	0.315
	*p*-value ^a^	0.421	0.421	0.679	
Catalase, CAT (U/mL)	Baseline	0.24 (0.15–0.39)	0.39 (0.34–0.71)	0.78 (0.56–0.83)	0.006 *
	EOT	0.90 (0.48–2.00)	1.82 (0.42–2.57)	1.52 (0.34–2.67)	0.796
	*p*-value ^a^	0.016 *	0.020 *	0.039 *	
Malondialdehyde, MDA (μmol/L)	Baseline	24.67 (18.37–29.11)	23.19 (14.67–28.37)	15.41 (10.59–26.89)	0.343
	EOT	15.41 (9.85–29.85)	17.26 (15.04–19.48)	12.44 (6.52–19.48)	0.185
	*p*-value ^a^	0.041 *	0.040 *	0.006 *	

Abbreviations: EOT, end of treatment (watercress consumption); SHS, non-smoker exposed to secondhand smoke; IQR, interquartile range. ^a^ The comparison between baseline data and EOT data in each group was analyzed by the Wilcoxon signed-rank test. ^b^ The comparison across three groups was analyzed by the Kruskal–Wallis test. * Significantly different (*p*-value < 0.05).

## Data Availability

The original contributions presented in this study are included in the article/Supplementary Materials. Further inquiries can be directed to the corresponding author.

## Supplementary Materials

The following supporting information can be downloaded at: https://www.mdpi.com/article/10.3390/antiox14121466/s1, Table S1: Exercise behavior across 7 days among SHS, Non-smoker, and Smoker groups; Table S2: Medication use across 7 days among SHS, Non-smoker, and Smoker groups.

## Abbreviations

The following abbreviations are used in this manuscript:

ABTS	2,2′-azino-bis-(3-ethylbenzothiazoline-6-sulfonic) acid
BMI	Body mass index
CAT	Catalase
CO	Carbon monoxide
COPD	Chronic obstructive pulmonary disease
EOT	End of treatment (watercress consumption)
FBG FDA	Fasting blood glucose Food and Drug Administration
FRAP	Ferric reducing antioxidant power
H_2_O_2_	Hydrogen peroxide
HDL-C	High-density lipoprotein cholesterol
HO^•^	Hydroxyl radical
IL-10	Interleukin-10
IL-1β	Interleukin-1 beta
IL-6	Interleukin-6
IQR	Interquartile range
ITCs	Isothiocyanates
LDL-C	Low-density lipoprotein cholesterol
MDA	Malondialdehyde
NCDs	Non-communicable diseases
NO^•^	Nitric oxide radical
O_2_^•−^	Superoxide anion radical
ROS	Reactive oxygen species
SHS	Non-smoker exposure to secondhand smoke
TAC	Total antioxidant capacity
TBA	Thiobarbituric acid
TC	Total cholesterol
TG	Triglyceride
TNF-α	Tumor necrosis factor-alpha
TPTZ	Triphenyltriazine
