# Supplementary material for: Impact of Watercress Consumption on Antioxidant Defense and Oxidative Stress Among Adults with Different Levels of Exposure to Cigarette Smoke in Chiang Mai, Thailand
Source: Antioxidants (Basel). 2025 Dec 7;14(12):1466. doi: 10.3390/antiox14121466 (PMC12730002; doi:10.3390/antiox14121466)
Supplement: Supplementary file 1 [file antioxidants-14-01466-s001.zip › antioxidants-3988804-supplementary.pdf]

Supplementary Materials

Analysis of association between watercress consumption and the covariates including exercise behavior and medication use

For the analysis of the association between watercress consumption and the covariates (exercise behavior and medication use), Fisher's exact test was performed using Stata software (version 17). No statistically significant associations were observed between watercress consumption and any of the covariates (P > 0.05), as presented in Supplementary Tables S1–S2.

Table S1. Exercise behavior across 7 days among SHS, Non-smoker, and Smoker groups

Watercress consumption (Day)		Non-smoker (n=15)		SHS (n=15)		Smoker (n=15)		P-values
		N	%	N	%	N	%	
Day 1								
	No	14	93.33	13	86.67	12	80.00	0.858
	Yes	1	6.67	2	13.33	3	20.00	
Day 2								
	No	13	86.67	13	86.67	14	93.33	1
	Yes	2	13.33	2	13.33	1	6.67	
Day 3								
	No	13	86.67	14	93.33	14	93.33	1
	Yes	2	13.33	1	6.67	1	6.67	
Day 4								
	No	12	80.00	14	93.33	14	93.33	0.594
	Yes	3	20.00	1	6.67	1	6.67	
Day 5								
	No	13	86.67	14	93.33	15	100.00	0.762
	Yes	2	13.33	1	6.67	0	0.00	
Day 6								
	No	13	86.67	13	86.67	13	86.67	1
	Yes	2	13.33	2	13.33	2	13.33	
Day 7								
	No	14	93.33	13	86.67	14	93.33	1
	Yes	1	6.67	2	13.33	1	6.67	

Abbreviations: SHS, non-smoker exposed to secondhand smoke

Table S2. Medication use across 7 days among SHS, Non-smoker, and Smoker groups

Watercress consumption (Day)		Non-smoker (n=15)		SHS (n=15)		Smoker (n=15)		P-values
		N	%	N	%	N	%	
Day 1								
	No	13	86.67	15	100.00	15	100.00	0.318
	Yes	2	13.33	0	0.00	0	0.00	
Day 2								
	No	14	93.33	15	100.00	15	100.00	1
	Yes	1	6.67	0	0.00	0	0.00	
Day 3								
	No	15	100.00	15	100.00	15	100.00	-
	Yes	0	0.00	0	0.00	0	0.00	
Day 4								
	No	15	100.00	15	100.00	15	100.00	-
	Yes	0	0.00	0	0.00	0	0.00	
Day 5								
	No	15	100.00	15	100.00	15	100.00	-
	Yes	0	0.00	0	0.00	0	0.00	
Day 6								
	No	14	93.33	15	100.00	14	93.33	1
	Yes	1	6.67	0	0.00	1	6.67	
Day 7								
	No	13	86.67	15	100.00	12	80.00	0.343
	Yes	2	13.33	0	0.00	3	20.00	

Abbreviations: SHS, non-smoker exposed to secondhand smoke
